# Hyaluronan Mediated Motility Receptor (HMMR) Encodes an Evolutionarily Conserved Homeostasis, Mitosis, and Meiosis Regulator Rather than a Hyaluronan Receptor

**DOI:** 10.3390/cells9040819

**Published:** 2020-03-28

**Authors:** Zhengcheng He, Lin Mei, Marisa Connell, Christopher A. Maxwell

**Affiliations:** 1 Department of Pediatrics, University of British Columbia, Vancouver, BC V5Z 4H4, Canada; zche@bcchr.ca (Z.H.); lmei@bcchr.ca (L.M.); marisa.connell@gmail.com (M.C.); 2 Michael Cuccione Childhood Cancer Research Program, BC Children’s Hospital, Vancouver, BC V5Z 4H4, Canada

**Keywords:** cell division, centrosome, hyaluronan, HMMR, multifunctional, RHAMM

## Abstract

Hyaluronan is an extracellular matrix component that absorbs water in tissues and engages cell surface receptors, like Cluster of Differentiation 44 (CD44), to promote cellular growth and movement. Consequently, CD44 demarks stem cells in normal tissues and tumor-initiating cells isolated from neoplastic tissues. Hyaluronan mediated motility receptor (HMMR, also known as RHAMM) is another one of few defined hyaluronan receptors. HMMR is also associated with neoplastic processes and its role in cancer progression is often attributed to hyaluronan-mediated signaling. But, HMMR is an intracellular, microtubule-associated, spindle assembly factor that localizes protein complexes to augment the activities of mitotic kinases, like polo-like kinase 1 and Aurora kinase A, and control dynein and kinesin motor activities. Expression of HMMR is elevated in cells prior to and during mitosis and tissues with detectable HMMR expression tend to be highly proliferative, including neoplastic tissues. Moreover, HMMR is a breast cancer susceptibility gene product. Here, we briefly review the associations between HMMR and tumorigenesis as well as the structure and evolution of HMMR, which identifies Hmmr-like gene products in several insect species that do not produce hyaluronan. This review supports the designation of HMMR as a homeostasis, mitosis, and meiosis regulator, and clarifies how its dysfunction may promote the tumorigenic process and cancer progression.

## 1. Introduction

Hyaluronan is an extracellular matrix component that absorbs water in tissues. Due to its hydrating nature, hyaluronan has many commercial uses, including cosmetic applications, but it also regulates the proliferation of certain stem cell populations and may enable certain hallmarks of cancer [1,2]. Hyaluronan receptors, such as Cluster of differentiation 44 (CD44), are highly expressed on stem cells in normal tissues and tumor-initiating cells isolated from neoplastic tissues [2,3,4]. Interestingly, one of few defined receptors for hyaluronan, termed hyaluronan mediated motility receptor (HMMR, also known as RHAMM, XRHAMM, IHABP, or CD168), is a centrosome and microtubule-associated protein that regulates cell growth. *HMMR* associates with breast cancer risk as well as cancer progression in multiple tumor types. Here, we outline the structure and evolution of HMMR, which supports its designation as a homeostasis, mitosis, and meiosis regulator rather than a hyaluronan receptor. This review helps to clarify how the perversion of HMMR function during cell division may support the tumorigenic process.

## 2. A Brief History of Hyaluronan Mediated Motility Receptor (HMMR)

HMMR was first identified as a constituent of a novel hyaluronan receptor complex purified from the supernatants of murine cells [5]. In 1992, a partial *Hmmr* transcript was cloned and the gene product was predicted to be about 58 kDa [5]. Many of the early experiments that described HMMR as a hyaluronan receptor used reagents generated against and/or recognizing proteins (56–75 kDa) that are now known to be much smaller than full-length HMMR (reviewed in [6]); we now know that murine *Hmmr* contains 18 exons and encodes a 95 kDa protein [6,7]. Often, experiments studied truncated *Hmmr* cDNA, which lack N-terminal sequence and key domains: for example, RHAMM 1v4, which only encodes exon 6–exon 18, and RHAMM2, which only encodes exon 10–exon 18 (reviewed in [6]). In 1999, five additional exons were cloned at the N-terminus of *HMMR*; as the gene product was found to be exclusively intracellular, it was suggested that it be renamed Intracellular hyaluronan binding protein (IHABP) [8,9]. This complicated history and the confusing nomenclature (i.e., RHAMM1, RHAMM1v4, RHAMM2, or IHABP) serves to cloud our understanding of the gene product’s function both as a microtubule-associated spindle assembly factor and as a putative hyaluronan receptor.

What’s in a name? That which we call a rose By any other word would smell as sweet.

*Romeo and Juliet*, 2, 2, 45-46

Juliet’s words are eloquent but the sharing of scientific information does rely upon accurate naming, classification, and ontology. Certain proteins assume unconventional roles during pathological conditions that may render their classification opaque [10,11]. However, an unbiased examination of the evolution of the *HMMR* gene and the gene product’s structure may provide clarity to the physiological functions and how those functions are perverted during the tumorigenic process.

## 3. The Conserved Basic C-Terminal Domain in HMMR Is a Leucine Zipper Motif

**Figure 1:**
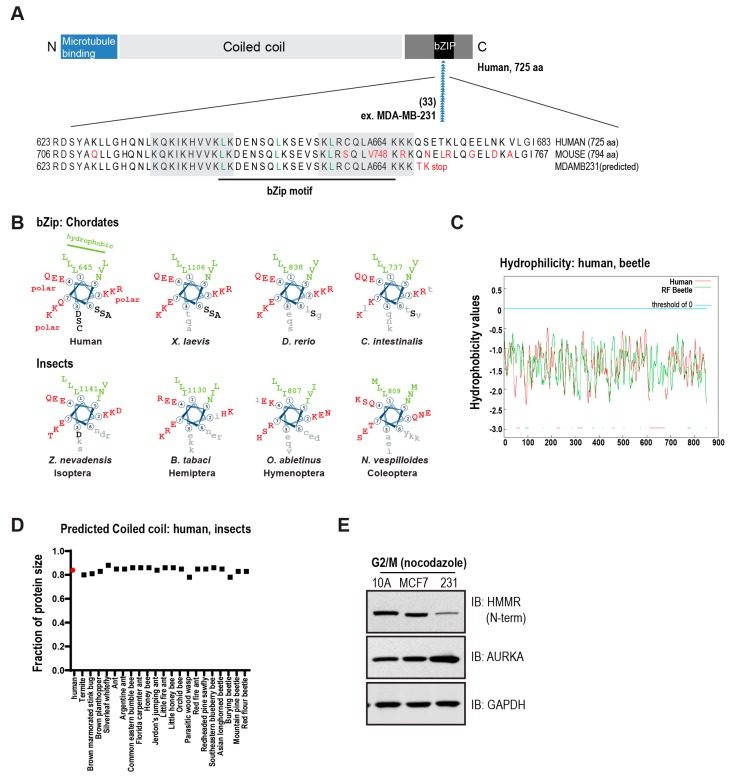
Conserved structural domains in chordate and insect Hyaluronan mediated motility receptor (HMMR). (**A**). The N-terminal microtubule-binding domain (blue) is separated from a C-terminal basic leucine zipper motif (bZIP, black) by a large predicted coiled-coil domain. The C-terminus targets the protein to the centrosome and regulates ubiquitination. Somatic mutations in HMMR occur at a hotspot in the bZIP motif and give rise to frameshift mutations that truncate or alter the C-terminus (ex. MDA-MB-231). The basic motifs in HMMR that interact with hyaluronan (HA) are boxed in grey. Conserved leucines are indicated in green for the bZIP motif, which is underlined in black. (**B**). Conservation of the bZip motif in vertebrate and invertebrate animals. Leucines (position 1) and hydrophobic residues (position 5) that comprise the hydrophobic face (green) are conserved. Polar residues (red) are also conserved while positions 3 and 6 show more variability (grey). (**C**). Hydrophobicity plots (AlignMe) comparing human HMMR (075330) with an Hmmr-like product in Red Flour beetle (D2A2B7, 813 aa). (**D**). Length represented as a fraction of total protein size for the predicted coiled coil length in human HMMR (red) and Hmmr-like gene products identified in insect species. (**E**). MDA-MB-231 cells contain an A^664^ frameshift mutation and express reduced levels of HMMR. The reduced expression for HMMR in MDA-MB-231 cells is not due to cell cycle distribution as levels are reduced in lysates from nocodazole-synchronized G2/M cells. Glyceraldehyde 3-phosphate dehydrogenase (GAPDH) serves as a loading control and Aurora kinase A (AURKA) serves as a control for the expression of a cell cycle-regulated gene product in the cell lysates.

To interact with hyaluronan, CD44 and other link module-based binding proteins use tandem repeat loops with homology to the cartilage link protein [12]. HMMR, however, was shown to interact with hyaluronan in an ionic manner through a basic, 35 amino acid, C-terminal region, which can be further subdivided into two motifs of 10 amino acids and 11 amino acids, respectively [13]. These motifs correspond with human HMMR amino acids 636–646 and amino acids 658–667, which are highly basic regions that became known as Basic (B) (X)7 Basic (B X7 B) motifs [13,14] (Figure 1A). Perhaps unsurprisingly, these basic residues in HMMR are essential for an ionic interaction with hyaluronan as well as heparin [13,14], and these interactions are abolished with increasing concentrations of salt [13].

It is important to note, however, the so-called “X7” residues are in fact highly ordered and evolutionarily conserved. When represented as a heptad repeat, this motif contains a leucine every seven residues (position 1) and conserved hydrophobic (interior) and polar (exterior) residues that enable a predicted coiled-coil structure and a basic leucine zipper (bZip) motif. Importantly, this bZip motif is conserved throughout chordates, including in the sea squirt *Ciona intestinalis*, as well as in species of insects (Figure 1B). That this highly basic stretch of amino acids binds in an ionic manner to hyaluronic acid in vitro is expected; that an urochordate and several species of insects encode a putative hyaluronan receptor (*Hmmr*) is remarkable, given these animals lack evidence for hyaluronan anabolism or synthesis, which first appears in amphioxus [15].

### 3.1. Structural Domains in HMMR

HMMR is predicted to be a hydrophilic protein (Figure 1C) and lacks a hydrophobic signal peptide or potential hydrophobic transmembrane domains. In fact, HMMR lacks the structural properties required for canonical extracellular export. Across chordate and insect species, HMMR is predicted to be a largely coiled-coil protein (Figure 1D, 80–85% of total amino acids) with microtubule binding domains at the N-terminus and a bZip motif [16] and degradation domains [17] at the C-terminus. These structural features—N-terminal microtubule binding, largely coiled-coil structure, C-terminal bZip motif—support its role as a non-motor spindle assembly factor.

The central region of human HMMR is a coiled-coil stalk (amino acids 69–681) that acts as a potential dimerization domain and binding region for other proteins. That is, HMMR interacts with CHICA/FAM83D through amino acids 365–546 [18], while amino acids 574–602 act as a calcium-dependent calmodulin binding domain [19]. Through its interaction with CHICA, HMMR locates dynein light chain 1 (DYNLL1) and CK1alpha to the spindle [20], and HMMR plays a similar role in docking BACH1 at the spindle [21], although the needed interaction domain is unknown. These protein complexes, formed through interactions with the coiled-coil stalk, play critical roles in the correct orientation of the mitotic spindle and the establishment of the cell division axis.

At the N-terminus, human HMMR possesses two microtubule binding subdomains at amino acids 40–59 and amino acids 76–90, respectively, with the latter domain encoded by exon 4 [19]. Through these domains, HMMR binds directly to microtubules [19]. While full-length HMMR is able to locate to centrosomes and bind to microtubules [16], the naturally occurring splice variant HMMR (-exon 4) localizes to the mitotic spindle during mitosis but shows nuclear localization during interphase [16,19]. Localization to the nucleus is also seen for a phosphorylated form of HMMR at Thr703 [22]. Phosphorylation at Thr703 was identified by phospho-proteomics [23] and, through the use of antibodies specific for phospho-Thr703 HMMR, it was revealed that phospho-Thr703 not only directs the protein to the nucleus but also may assist in the regulation of the Ran-dependent nuclear transport of targeting protein for Xklp2 (TPX2) [22].

The very N-terminal residues in HMMR are uniquely similar to those of mira/Miranda [24], a determinant of asymmetric neuroblast divisions in *Drosophila*. Miranda is a docking protein in neuroblasts that is responsible for the asymmetric cortical localization of key proteins and mRNAs that regulate neuroblast cell fate, including Prospero and brain tumor (Brat) [25,26]. Despite this critical role in *Drosophila* neurodevelopment, Miranda has no known orthologs outside the order Diptera. Importantly, Miranda and HMMR share significant similarity in their very N-terminal sequence [24]. These N-terminal residues in Miranda are needed for microtubule-dependent asymmetric localization [27]. Moreover, both Miranda and HMMR are largely coiled-coil proteins that bind microtubules but HMMR has not yet been determined to play an orthologous function during asymmetric cell divisions.

The conserved, C-terminal bZip motif in HMMR overlaps with the designated B-X7-B motifs, which bind in an ionic manner to hyaluronan and heparin [13,14]. The bZip motif of HMMR contains 72% homology with a C-terminal bZip motif in kinesin-like protein Kif15/hKlp2 (amino acids 1342–1388) [16]. Importantly, the bZip motif in hKlp2 locates the protein to microtubule minus ends through an interaction with targeting protein for Xklp2 (TPX2) [28]. While TPX2 was first defined by complexing with the bZip motif in hKlp2 and targeting the complex to microtubule minus ends [28,29], TPX2 has now been extensively characterized as both a critical activator for the mitotic kinase Aurora kinase A as well as other functions (reviewed in [30]). For HMMR, the bZip motif locates the protein to the centrosome and HMMR complexes with a significant fraction of TPX2 in a cell cycle-dependent manner [31,32,33,34]. These complexes serve to both regulate mitotic kinase activities [33] and alter Eg5 motor protein processivity [35,36].

Taken together, the structure of HMMR is one of a largely coiled-coil microtubule-associated protein, which can bind to microtubules directly through its N-terminus and localize to the centrosome through its C-terminal bZip motif. HMMR serves as a binding partner for spindle assembly factors, such as TPX2, DYNLL1, and CHICA/FAM83D, to regulate the assembly, stability and positioning of spindle microtubules during mitosis and meiosis.

### 3.2. Evolution of HMMR

The presence of *hmmr* orthologs amongst invertebrate animals has not been examined likely because arthropods do not express hyaluronan [37]. By using the conserved bZip motif as a landmark, we identified putative *hmmr* orthologs in many, but not all, orders of insects (Table S1). The bZip motif in *hmmr* homologs are most highly conserved in Isoptera, the earliest insect lineage represented (Figure 1B). We found no evidence for *hmmr* homologs in genomes in the order Diptera, which includes *Drosophila*.

We discovered that *hmmr* and *mira* are mutually exclusive amongst insect species with *mira* encoded exclusively in Dipterans. Comparison of N-terminal homology regions [24] from 127 known and predicted Miranda and HMMR/Hmmr sequences identified conservation in critical residues (Ala6 and Ala18, [24]), suggesting that *mira* potentially evolved in Diptera from an ancestral *hmmr* (Table S1). 

It is a striking finding that a defined receptor (*Hmmr*) is encoded within multiple species that do not produce its putative ligand (hyaluronan). As *Hmmr*-like orthologs are encoded in species that predate the first appearance of hyaluronan by millions of years (in amphioxus [15]), we must conclude that Hmmr did not originally evolve as a hyaluronan receptor.

### 3.3. Conserved HMMR-NudC Domain-Containing Protein 2 (NUDCD2) Gene Cluster

**Figure 2:**
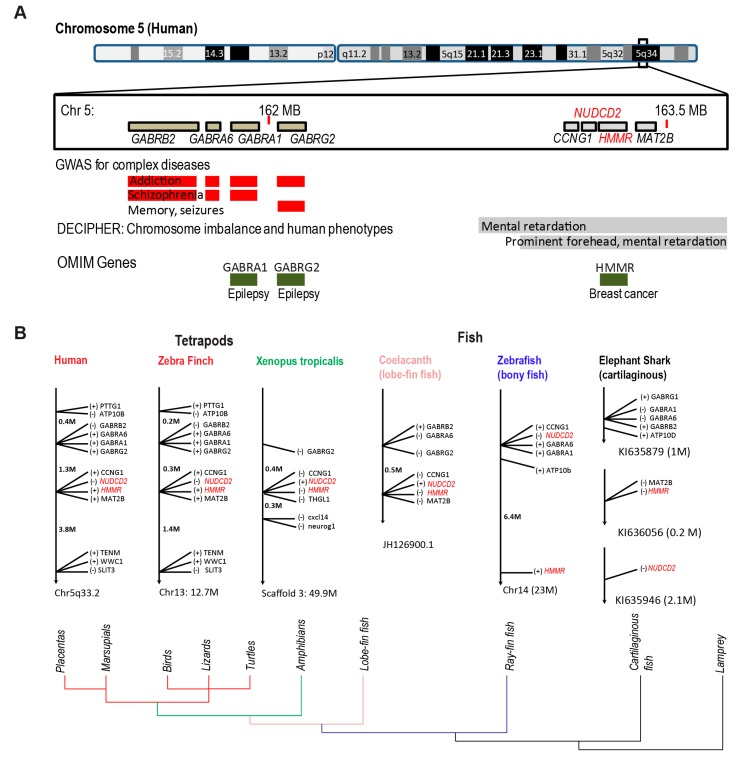
Conserved *HMMR-NUDC domain-containing protein 2 (NUDCD2)* gene cluster in tetrapods. (**A**). Chromosome location for the *CCNG1-NUDCD2-HMMR* and *GABAA* gene clusters on human chromosome 5q33.1. Importantly, *NUDCD2* and *HMMR* are oriented in opposite directions such that their 5′-ends are separated by only 530 base-pairs. The proximity of these gene clusters is conserved throughout chordates. In humans, this region of chromosome 5q associates with numerous human diseases and phenotypes related to neural development and homeostasis. (**B**). Chromosome locations for the *CCNG1-NUDCD2-HMMR* and *GABAA* gene clusters shows conservation of clusters within tetrapod species and lobe-fin fish species. The orientation of genes is indicated as forward (+) or reverse (−).

The appearance of HMMR in multiple species that lack hyaluronan strongly argues against it playing an evolutionarily-conserved role in hyaluronan binding. Indeed, the conserved gene location of *HMMR* across multiple species of tetrapods and fish may implicate an evolutionarily-conserved role related to cell cycle and dynein motor activity. That is, *HMMR* is found in a small four gene cluster, along with *NUDCD2* (NudC domain-containing protein 2)*, CCNG1* (cyclin G1), and *MAT2B* (methionine adenosyltransferase 2B), at 163.5 MB of human chromosome 5q34 (Figure 2A).

The *HMMR-NUDCD2* gene cluster is proximal to a cluster of gamma-aminobutyric acid (GABA) A receptor subunit genes at 162 MB of human chromosome 5q33.3; this region of human chromosome 5q associates with human diseases related to neural development and/or homeostasis, including addiction, schizophrenia, memory, seizures (for 5q33.1), prominent forehead, and mental retardation (for 5q34). The proximity of these two small gene clusters has been maintained through the evolution of jawed vertebrates (Figure 2B): one cluster encodes cell cycle and dynein related gene products (*CCNG1-NUDCD2-HMMR*) while the other encodes GABAA receptor subunits.

In cartilaginous fish and *Ciona*, which is potentially the closest invertebrate to humans, *HMMR*, *NUDCD2*, *GABRB2*, and *CCNG1* are located at different chromosomal sites (Figure 2B). Initiating with bony fish, however, *NUDCD2* and *CCNG1* are clustered with *GABRA1* and *GABRA6* while *HMMR* is located relatively proximal on chromosome 14 (Figure 2B). In lobe-fin fish, the two clusters separate and their organization and overall structure are maintained through tetrapod evolution (Figure 2B).

It is important to note the conserved orientation of *HMMR* and *NUDCD2* in these small gene clusters. That is, the genes are oriented in opposite directions; for example, on human chromosome 5q, *HMMR* is in a forward orientation (163,460 kB–163,492 kB) and *NUDCD2* is in a reverse orientation (163,446 kB–163,460 kB) so that the 5′ ends of the genes are 530 base-pairs apart. This conserved, close proximity of *NUDCD2-HMMR* may be a chance of nature. But, importantly, *HMMR* and *NUDCD2* perform similar cell cycle-regulated functions as adaptors for dynein motor proteins and each have critical roles in the process of neural development and homeostasis.

## 4. HMMR Functions as a Homeostasis, Mitosis, and Meiosis Regulator

### 4.1. HMMR Is Needed for Tissue Homeostasis and Neural Development

*HMMR* is expressed in the developing nervous system [38] as well as in the proliferative regions of the adult mouse brain [39]. The mutation or loss of *HMMR* in vertebrate animal models induces neurodevelopmental defects [7,40,41]. *Hmmr^tm1a/tm1a^* mice encode *Hmmr* with targeted disruption following exon 2 while *Hmmr^m/m^* mice encode *Hmmr* that potentially retain N-terminal HMMR structure due to targeted disruption after exon 10. As a consequence, loss-of *Hmmr* phenotypes differ in these animals. *Hmmr^tm1a/tm1a^* mice suffer neonatal lethality and display heterogeneous cortical sizes, enlarged ventricles, and alterations in neural cell subsets [7]. However, *Hmmr^m/m^* mice are viable but, *Hmmr^m/m^* embryos undergo transient megalencephaly during development [40]. In both mouse models (*Hmmr^tm1a/tm1a^* mice and *Hmmr^m/m^* mice), the C-terminus of *Hmmr* is required to orient neural stem cell divisions [7,40].

A third animal model, *Xenopus* embryos treated with morpholinos targeting *hmmr*, also present with neurodevelopmental defects. *Hmmr* morphant embryos develop brain defects, including defective neural tube closure, narrowed forebrains, loss of hemispheric separation, and smaller olfactory bulbs [41]. In this model, *hmmr* morphant cells rescued with a mutant lacking the N-terminal 130 amino acids were rounded and displayed web-like microtubule arrays rather than the linear arrays observed in wild-type neural cells [41]. Thus, the N-terminus of Hmmr was required for the polarization of cells in the deep neural layer.

Loss of *Hmmr/hmmr* phenotypes are reminiscent, but not overlapping with those seen for LIS1 (Lissencephaly 1), an alternate cytoplasmic dynein partner protein; classical lissencephaly, a brain developmental disease characterized by decreased cortical complexity and generally larger brain size, results from mutations in the *LIS1* gene [42]. A complex between dynein and the coiled-coil adaptor proteins LIS1, NUDC (nuclear distribution C, dynein complex regulator) and NUDE-like 1 (NDEL1) is required for correct corticogenesis. The loss of this complex, mediated through *LIS1* mutations, can result in a cortical malformation disorder associated with severe cognitive impairment and epilepsy [43].

Importantly, NDEL1 relies upon post-translational modification by Aurora kinase A and TPX2 to modify microtubule dynamics, neuronal migration and neurite extension downstream [44,45,46]. Provocatively, NUDCD2 also regulates the LIS1-dynein complex [47], centrosome function [48], and mitotic spindle integrity [49]. Thus, the evolutionally conserved *HMMR-NUDCD2* gene cluster encodes two coiled-coil centrosome proteins that complex with dynein and play critical roles in homeostasis, centrosome function, and mitotic spindle integrity.

In mouse models, the mutation or loss of *HMMR/Hmmr* expression also disrupts the correct development or homeostasis of gonadal tissues [7,50,51]. While the mechanisms responsible are yet to be determined, these phenotypes are likely reflective of the high expression of *HMMR* in gonadal tissues and the necessary role played by dynein, and its adaptor proteins, during the processes of gametogenesis and spermatogenesis [52,53,54].

### 4.2. HMMR Regulates Spindle Assembly in Mitotic Cells and Meiotic Extracts.

HMMR is a largely coiled-coil protein that can bind to microtubules directly through its N-terminus [19] and localize to the centrosome through a C-terminal bZip motif [16], which is structurally very similar to the C-terminal bZip motif in Xklp2 that enables an interaction with TPX2 [16,28]. The similarity in these domain structures seeded the hypothesis that HMMR also interacts with TPX2, which has been confirmed experimentally through the study of HMMR/RHAMM in human mitotic cells [16,33,35] and Xenopus RHAMM (XRHAMM) in *Xenopus* meiotic extracts [32,34,36,55].

The interaction between HMMR and TPX2 is important for Ran-dependent microtubule assembly near chromosomes [32,34], the activity of Aurora kinase A and microtubule nucleation at spindle poles [33], and the regulation of the processivity of the kinesin motor Eg5 [35,36]. The C-terminal bZip motif in Xklp2 regulates its location to microtubule minus ends in a dynein-dependent manner [28]; similarly, the C-terminal bZip motif in HMMR regulates its location to centrosomes and interaction with dynein [31].

HMMR is critical for the orientation of the mitotic spindle in human mitotic cells. HMMR locates DYNLL1 indirectly through an interaction with CHICA/FAM83D [18], which serves to modulate the position of the mitotic spindle through three complementary mechanisms: 1. HMMR localizes DYNLL1 at spindle poles, which dampens local dynein motor activities when brought proximal to the cell cortex [18]; 2. HMMR modulates the local activity of PLK1 and locates Ran-GTP to the spindle pole [7]; and, HMMR binds CHICA/FAM83D to locate and activate the protein kinase CK1alpha [20].

Each of the preceding mitotic and meiotic functions were predicted by the domain structure of HMMR. In addition to such hypothesis-based studies, a number of non-biased, genome-wide analyses also indicate similar mitotic roles of HMMR. The All RNA-Seq and Chip-Seq Sample and Signature Search (ARCHS4) tool, which mines sequencing data from 103,083 mouse and 84,863 human samples [56], predicts HMMR functions in processes related to mitosis and chromosome segregation, kinetochore, and nuclear pore complex assembly, which have all been independently and experimentally validated [16,22,35]. Using this tool [56], *HMMR* is found to be co-expressed with *TOP2A, KIF11, TPX2, ECT2, BUB1, KIF20A, NUSAP1, KIF20B, SMC2*, and *CCNA2*; each of these gene products are involved with cell cycle regulation, spindle organization, and chromosome segregation. A complementary proteome-wide analysis of mitotic substages, which combined specific intracellular immunolabeling protocols and FACS separation of interphase and mitotic cells, identified HMMR as one of 14 proteins that peak in abundance in G2-phase and mitosis along with other cell cycle gene products, such as Aurora A and B, Polo-like kinase 1, and CENPF [57]. Finally, the Mitocheck consortium combined several powerful screening tools, such as RNA interference, time-lapse microscopy and computational image processing that assessed chromosome and nuclear morphology, to profile the roles of ~21,000 gene products during mitosis [58]. HMMR depletion was associated with mitosis-specific phenotypes, such as strange nuclear shape, polylobed nuclei and chromosome segregation errors, leading to *HMMR* being designated a validated mitotic hit [58].

Given the critical mitotic functions of HMMR, it is unsurprising that *HMMR* is predicted to be regulated by proliferation associated transcription factors, FOXM1, E2F4, and MYC [56] and, experimentally, by YAP-TEAD of the Hippo pathway [59]. Similarly, it is unsurprising that *HMMR* expression is transcriptionally downregulated by the tumor suppressor TP53 [60]. Perhaps less predictable are the mechanisms utilized for post-transcriptional regulation of HMMR expression. Along with Bard1 and other Ran-dependent spindle assembly factors, Hmmr was identified as a substrate for the ubiquitin ligase anaphase-promoting complex/cyclosome (APC/C) [17]; the recognition and proteolysis of Hmmr and Bard1 (as well as complexed Brca1) relied upon APC/C recognition motifs, such as destruction (D) box, KEN box, or TEK box [17]. For Hmmr, D, KEN, and TEK boxes were identified in the C-terminal domain [17]. HMMR is also a substrate for the BRCA1/BARD1 E3 ubiquitin ligase [61] and deficient BRCA1 function leads to the stabilization of HMMR [61,62]. The HMMR-BRCA1 interaction influences mammary tumorigenesis as indicated by the association between *HMMR* polymorphisms and breast cancer risk in carriers of *BRCA1* mutations but not *BRCA2* mutations [63]. Similarly, a HMMR partner protein, DYNLL1, has been correlated with progression free survival in carriers of *BRCA1* mutations but not *BRCA2* mutations and shown to modulate PARP inhibitor sensitivity in BRCA1-mutant cells [64]. The potential importance of HMMR-DYNLL1 mediated control of the cell division axis in the context of *BRCA1* mutations is not yet known but may help to explain how *BRCA1* mutations perturb the differentiation hierarchy present in the normal human mammary gland [65,66,67].

## 5. HMMR Associates with Breast Cancer risk, Cancer Prognosis, and Progression

The expression of HMMR is cell cycle-regulated with peak expression between late G2 phase and early mitosis [31]. Consistently, *HMMR* expression is low in most healthy tissues but is elevated in proliferative tissues, such as the testis, spleen, placenta, and thymus [7,8]. Moreover, elevated *HMMR* expression associates with poor prognosis in a variety of cancers, such as breast cancer [68], colorectal cancer [69], stomach cancer [70], endometrial cancer [71], prostate cancer [72], and multiple myeloma [73]. But, the association between HMMR and cancer is not as simple as high *HMMR* expression demarcates very mitotic tumors, which correlates with more aggressive tumor growth and poor patient survival.

For several types of cancers, poor patient survival also associates with low *HMMR* expression. For example, hemizygous deletion of *HMMR* is present in almost half of malignant peripheral nerve sheath tumors [74,75]; *HMMR* expression is diminished in 96% of human seminomas [50]; and, germline *HMMR* variants that associate with lower expression also associate with increased breast cancer risk in some patient cohorts [61,76,77]. Moreover, an *HMMR* splice variant that loses microtubule binding activity is expressed at an elevated level in a variety of cancers. That is, the N-terminal domain of HMMR binds microtubules, encoded in part by exon 4 [16,19], and the expression of a naturally occurring splice variant that lacks exon 4 (-exon 4) correlates with progression of multiple myeloma [73] and breast cancers [78]. *HMMR* (-exon 4) expression is also sufficient to promote pancreatic islet tumor growth and metastasis to lymph nodes and liver [79]. As indicated by the cancer cell-line encyclopedia and COSMIC databases, *HMMR* contains a mutational hotspot encoded by a homopolymeric adenine tract within the C-terminal basic leucine zipper motif (Figure 1A). Indeed, immortal MDA-MB-231 breast cancer cells contain an A^664^ frameshift mutation (cDNA change c.1992_1993insA), and the expression of HMMR is greatly reduced in lysates from nocodazole-synchronized, G2/M phase lysates generated from MDA-MB-231 cells relative to MCF7 cells or MCF10A cells (Figure 1E). Thus, aggressive cancers may contain either abnormally high or abnormally low expression of HMMR, and tumors are frequently characterized by the expression of a putative loss of function *HMMR* splice variant (-exon 4) or frameshift mutations in the homopolymeric adenine tract within the C-terminal bZip motif (Figure 1A).

*HMMR* is a low penetrance breast cancer susceptibility gene [61]; genotyping of three *HMMR* haplotype-tagging single nucleotide polymorphisms (htSNPs) identified statistically significant associations with risk of breast cancer [61]. Importantly, *HMMR* htSNPs showed an association with either germline overexpression (rs10515860 SNP; A-C-A haplotype) or germline downregulation of *HMMR* (G-C-A-T-G haplotype) [61]. HMMR is also a key substrate for the BRCA1-BARD1 E3 ubiquitin ligase during the process of spindle assembly [61]; dysfunction of BRCA1 is well-documented to stabilize HMMR protein expression [50,55,61,62]. Moreover, common *HMMR* variants modify the risk of developing breast cancer for carriers of *BRCA1*, but not *BRCA2*, mutations [63]. Thus, there is evidence to support the hypothesis that HMMR protein abundance must be tightly regulated through transcriptional control by TP53 [60] and the Hippo pathway [59] and post-translational turnover by BRCA1-BARD1 [61] and the anaphase promoting complex [17]. Consistent with this, silencing or elevating *HMMR* expression disrupts microtubule-based processes during cell division, and results in mitotic spindle abnormalities, genome instability, and changes to the cell division axis and progenitor cell fate.

## 6. Conclusions

It is also possible, and has not been disproven, that the unconventional export of HMMR during pathological states may enable non-physiological functionalities, such as ionic interaction between the C-terminal bZip motif and acidic hyaluronan or heparin. However, *hmmr* is encoded in numerous species that lack hyaluronan, which argues strongly that HMMR did not evolve as a hyaluronan receptor; rather, the evolution of *HMMR* in a small gene cluster with the dynein adaptor *NUDCD2* and the structure of the HMMR gene product support the designation of HMMR as a homeostasis, mitosis, and meiosis regulator. Indeed, HMMR is a largely coiled coil gene product with evolutionarily conserved N-terminal and C-terminal domains that target the protein to microtubules and centrosomes, respectively. Consistent with these structural features defining the protein’s physiological function, recent studies indicate necessary roles for HMMR during cell division and microtubule-associated processes related to neural development, control of the cell division axis, and progenitor cell fate in three different vertebrate animal models. 

## Supplementary Materials

The following are available online at https://www.mdpi.com/2073-4409/9/4/819/s1, Table S1: HMMR and Miranda sequences and domains in insect species.
