# Supplementary material for: Hyaluronan Mediated Motility Receptor (HMMR) Encodes an Evolutionarily Conserved Homeostasis, Mitosis, and Meiosis Regulator Rather than a Hyaluronan Receptor
Source: Cells. 2020 Mar 28;9(4):819. doi: 10.3390/cells9040819 (PMC7226759; doi:10.3390/cells9040819)
Supplement: Supplementary file 1 [file cells-09-00819-s001.pdf]

Table S1: HMMR and Miranda sequences and domains in insect species. Predicted gene product names (indicated by an asterix) were produced from the NCBI eukaryotic gene prediction tool, Gnomon. The coiled coil was predicted using NPS@ prediction using a 21-residue window although 14- and 28- residue windows demonstrated similar results.

Common name	Species; Order	Pubmed ID Name, *predicted	# of residues	N-terminal Domain (20 aa)	Coiled coil (Residues) % total	Bzip domain
Human	*H. sapiens; Primates*	NP_001136028.1 HMMR	725	MSFPKAPLKRFNDPSGCAPS	(73-679) 84%	+
Mouse	*Mus musculus*	AAH21427.1 Hmmr	836	MSFPKAPLKRFNDPSGCAPS	(82-804) 86%	+
African clawed frog	*Xenopus laevis*	NP_001087936.1 Hmmr	1175	MSFPKAPLKRFNEHIGCAPA	(90-1146) 89%	+
Termite	*Z. nevadensis; Isoptera*	XP_021929154.1 *Hmmr	1219	MSFPRAKIQRFNDATSCAPP	(148-1125) 80%	+
Brown marmorated stink bug	*H. halys; Hemiptera*	XP_014283477.1 *Hmmr	1241	MSFPKARILRFNEEMTCAPP	(145-1150) 81%	+
Brown planthopper	*N. lugens; Hemiptera*	XP_022188703.1 *Hmmr	1107	MSFPKAKLHRFNEEMTCAPP	(111-1031) 83%	+
Silverleaf whitefly	*B. tabaci; Hemiptera*	XP_018903570.1 *Hmmr	1198	MSFAKAKIHRFNDDVSCAPP	(111-1160) 88%	+
Green peach aphid	*M. persicae; Hemiptera*	XP_022179692.1 *Hmmr	382	MSFVKSKIKRFNEVSSCAPP	(106-324) 57%	No
Pea aphid	*A. pisum; Hemiptera*	XP_001945179.1 *Hmmr	382	MSFVKSKIKRFNEVSSCAPP	(106-323) 57%	No
Russian wheat aphid	*D. noxia; Hemiptera*	XP_015371136.1 *Hmmr	381	MSFVKSKIKRFNEVSSCAPP	(106-322) 57%	No
Ant	*D. quadriceps; Hymenoptera*	XP_014470916.1 *Hmmr	1011	MSFSKAKIQRFNEIGSEAPP	(122-979) 85%	+
Argentine ant	*L. humile; Hymenoptera*	XP_012229057.1 *Hmmr	1011	MSFSKAKIQRFNELGSEAPP	(110-974) 85%	+
Common eastern bumble bee	*B. impatiens; Hymenoptera*	XP_003491759.1 *Hmmr	1008	MSFSKARIQRFNEFENDVPP	(108-973) 86%	+
Florida carpenter ant	*C. floridanus; Hymenoptera*	XP_011266101.1 *Hmmr	1009	MSFSKARIQRFNELGSEAPP	(109-977) 86%	+
Honey bee	*A. melifera; Hymenoptera*	XP_001121644.2 *Hmmr	1000	MSFSKARIQRFNEFENDVPP	(108-968) 86%	+
Jerdon's jumping ant	*H. saltator; Hymenoptera*	XP_011151919.1 *Hmmr	1007	MSFSKARIQRFNEMGSEAPP	(124-969) 84%	+
Little fire ant	*W.auropunctata; Hymenoptera*	XP_011698344.1 *Hmmr	1010	MSFSKARIQRFNEFASDAPP	(107-980) 86%	+
Little honey bee	*A. florea; Hymenoptera*	XP_012350052.1 *Hmmr	999	MSFSKARIQRFNEFENDVPP	(108-967) 86%	+
Orchid bee	*E. mexicana; Hymenoptera*	XP_017764489.1 *paramyosin-like	1027	MSFSKARIQRFNECENDVPP	(109-985) 85%	+
Parasitic wood wasp	*O. abietinus; Hymenoptera*	XP_012282310.1 *Hmmr	875	MSFSKARIQRFNELGSEAPP	(108-790) 78%	+
Red fire ant	*S. invicta; Hymenoptera*	XP_011176081.1 *Hmmr	673	MSFSKARIQRFNELGSDAPP	(68-642) 85%	+
Redheaded pine sawfly	*N. lecontei; Hymenoptera*	XP_015517286.1 *Hmmr	864	MSFPRARIQRFNEFSNDVPP	(108-844) 85%	+

Southeastern blueberry bee	*H. laboriosa; Hymenoptera*	XP_017797889.1 *Hmmr	1009	MSFSKARIQRFNEFENDVPP	(111-977) 86%	+
Asian longhorned beetle	*A. glabripennis; Coleoptera*	XP_018571601.1 *Hmmr	863	MSFSKAKIQEFNDIKPIAPG	(100-834) 85%	+
Burying beetle	*N. vespilloides; Coleoptera*	XP_017783900.1 *Hmmr	885	MSFSRAKLTRFNEKLPCTPS	(100-787) 78%	+
Mountain pine beetle	*D. ponderosae; Coleoptera*	XP_019761265.1 *polyamine-modulated factor 1-binding protein 1-like	648	MSFSKAKIIRFNDVNGNSKP	(74-613) 83%	No
Red flour beetle	*T. castaneum; Coleoptera*	XP_008191709 *Hmmr	813	MSFSKAKIQRFNDVKECTPS	(102-780) 83%	+

**Common name**	**Species; Order**	**Pubmed ID Name, *prediction**	**# of residues**	**N-terminal Domain (20 aa)**	**Coiled coil (Residues) % total**	**Bzip domain**	**Homology to Mira**
American malaria mosquito	*A.darlingi; Diptera*	ETN62704.1 Miranda	885	MSFSKAKLKRFNDVPVVSSP	(129-752) 70%	No	
Fruit fly	*D. melanogaster; Diptera*	NP_477291.1 Miranda	829	MSFSKAKLKRFNDVDVAICG	(159-699) 65%	No	
Fruit fly	*D. erecta; Diptera*	XP_015010478.1 (uncharacterized)	795	MSFSKAKLKRFNDVDTVACG	(154-664) 64%	No	62%
Green bottle fly	*L. cuprina; Diptera*	KNC26637.1 (hypothetical)	858	MSFSKAKLKRFNDIDVAACG	(156-726) 66%	No	62%
House fly	*M. domestica; Diptera*	XP_005182574.1 *Uso1	830	MSFSKAKLKRFNDVDTVACG	(157-701) 66%	No	62%
Mediterranean fruit fly	*C. capitata; Diptera*	XP_004519307.1 *A-kinase anchor 9	841	MSFSKAKLKRFNDVDVECAN	(164-709) 65%	No	57%
Midge	*C.marinus; Diptera*	CRL00824.1 (unknown)	673	MSFSKAKLKRFNDITDFASP	(115-580) 69%	No	19%
Snowberry fruit fly	*R. zephyria; Diptera*	XP_017469429.1 *unconventional myosin XV11a isoformX1	884	MSFSKAKLKRFNDVDVDCAN	(174-747) 65%	No	60%
Solanum fruit fly	*B. latifrons; Diptera*	XP_018802054.1 *trichohyalin isoform X1	873	MSFSKAKLKRFNDVDVDCAN	(164-739) 66%	No	60%
Yellow fever mosquito	*A. aegypti; Diptera*	XP_021707224.1 *Ddb, leucine-rich repeat containing protein	950	MFSKAKLKRFNDIPVVSSPS	(119-894) 82%	No	65% Anopheles
